# Knowledge, Perceptions and Attitude of Researchers Towards Using ChatGPT in Research

**DOI:** 10.1007/s10916-024-02044-4

**Published:** 2024-02-27

**Authors:** Ahmed Samir Abdelhafiz, Asmaa Ali, Ayman Mohamed Maaly, Hany Hassan Ziady, Eman Anwar Sultan, Mohamed Anwar Mahgoub

**Affiliations:** 1 Department of Clinical pathology, National Cancer Institute, Cairo University, Kasr Al-Aini Street, from Elkhalig Square, Cairo, 11796 Egypt; 2 Department of Pulmonary Medicine, Abbassia Chest Hospital, Ministry of Health and Population, Cairo, Egypt; 3 Department of Anaesthesia and Surgical Intensive Care, Faculty of Medicine, Alexandria University, Alexandria, Egypt; 4 Department of Community Medicine, Faculty of Medicine, Alexandria University, Alexandria, Egypt; 5 Department of Microbiology, High Institute of Public Health, Alexandria University, Alexandria, Egypt

**Keywords:** ChatGPT, Artificial Intelligence, ELSI, Academic Research, Chatbots

## Abstract

**Introduction:**

ChatGPT, a recently released chatbot from OpenAI, has found applications in various aspects of life, including academic research. This study investigated the knowledge, perceptions, and attitudes of researchers towards using ChatGPT and other chatbots in academic research.

**Methods:**

A pre-designed, self-administered survey using Google Forms was employed to conduct the study. The questionnaire assessed participants’ knowledge of ChatGPT and other chatbots, their awareness of current chatbot and artificial intelligence (AI) applications, and their attitudes towards ChatGPT and its potential research uses.

**Results:**

Two hundred researchers participated in the survey. A majority were female (57.5%), and over two-thirds belonged to the medical field (68%). While 67% had heard of ChatGPT, only 11.5% had employed it in their research, primarily for rephrasing paragraphs and finding references. Interestingly, over one-third supported the notion of listing ChatGPT as an author in scientific publications. Concerns emerged regarding AI’s potential to automate researcher tasks, particularly in language editing, statistics, and data analysis. Additionally, roughly half expressed ethical concerns about using AI applications in scientific research.

**Conclusion:**

The increasing use of chatbots in academic research necessitates thoughtful regulation that balances potential benefits with inherent limitations and potential risks. Chatbots should not be considered authors of scientific publications but rather assistants to researchers during manuscript preparation and review. Researchers should be equipped with proper training to utilize chatbots and other AI tools effectively and ethically.

**Supplementary Information:**

The online version contains supplementary material available at 10.1007/s10916-024-02044-4.

## Introduction

ChatGPT, the OpenAI chatbot released in November 2022, has ignited significant academic and media interest. While chatbot technology existed before, recent advances in AI, driven by substantial resource investment, have ushered in a paradigm shift. ChatGPT leverages advanced deep learning techniques and extensive data training to generate human-like responses to user inputs [1]. The OpenAI website describes it as capable of engaging in dialogue, answering follow-up questions, recognizing errors, and rejecting inappropriate requests [2].

With over 100 million users in its first two months, excitement is brewing around ChatGPT’s potential across diverse societal domains, encompassing academia, commerce, professional settings, and personal life [4]. A multitude of applications are being explored, spanning marketing, advertising, consulting, customer service, and finance [10]. ChatGPT’s transformative potential lies in its ability to enhance data accuracy and analysis, support diverse languages, and automate repetitive tasks [5].

While ChatGPT shares a lineage with previous AI models in terms of its language processing architecture and deep learning training methodology, its distinguishing feature lies in its open accessibility as a conversational chatbot. The underlying architecture, GPT-3, released in 2020, exhibited impressive capabilities for generating human-like text, writing essays, translating and summarizing lengthy texts, and answering complex questions [6]. However, ChatGPT differentiates itself by prioritizing conversational interaction, enabling users to engage in open-ended dialogue and receive dynamic responses tailored to their specific queries and context. While GPT-3 boasts a significantly larger parameter count of 175 billion compared to ChatGPT’s 20 billion, the latter exhibits specialized optimization for human-like conversational text generation. This design choice, in conjunction with its open availability and accessibility, has fueled ChatGPT’s rapid global dissemination and swift rise in popularity [7].

ChatGPT’s emergence has precipitated a surge of media, social media, and academic discourse, prompting calls for reevaluating academic practices considering its capabilities. Notably, demonstrations have shown ChatGPT’s ability to convincingly generate facsimiles of research abstracts, even deceiving some scientists [8]. An early example of ChatGPT’s academic application can be found in an article published in February 2023 [9]. While the generated text demonstrated impressive fluency and minimal grammatical errors, showcasing clarity through its use of simple language, it revealed a certain superficiality and robotic quality. Notably, the text lacked the depth of analysis and stylistic diversity typically associated with human authorship. Nevertheless, advancements in ChatGPT’s training are anticipated to mitigate these limitations in the near future [9].

ChatGPT’s capabilities extend beyond mere text generation, as demonstrated by its successful integration into the drafting of editorials and preprints that bypassed plagiarism detection measures [10–12]. However, within the dynamic landscape of scientific knowledge, it is imperative to leverage technological advancements while safeguarding the essential human element. The software’s potential for rapid and accurate output offers the prospect of enhancing efficiency and freeing researchers to engage in more critical tasks [13]. This confluence of factors has catalyzed calls for reimagining the research process. Our present study aims to investigate the knowledge, perceptions, and attitudes of Egyptian researchers towards the utilization of ChatGPT and other chatbots within the research context.

## Methods

### Study Design and Target Population

The present study employed a cross-sectional design, conducted entirely online. Our target population encompassed researchers affiliated with diverse universities and academic institutions across Egypt. To ensure a representative and heterogeneous sample, we implemented a multi-pronged approach recruitment strategy to identify, then to contact potential participants.

### Leveraging Online Research Networks

- Targeted Searches on Scholarly Platforms: We utilized keyword-based searches on Google Scholar, Microsoft Teams, and ResearchGate to identify researchers actively publishing in fields relevant to the study. We then filtered results based on institutional affiliation and research focus to curate a targeted pool of potential participants.
- Professional Social Media Groups: We disseminated study invitations and concise descriptions within pertinent research discussion groups on platforms like Facebook and LinkedIn. These communities often require academic credentials or institutional affiliation for membership, enhancing the likelihood of reaching qualified researchers.

### Collaboration with Universities and Research Institutions

- Faculty Liaison and Research Office Outreach: We established contact with some key individuals, such as faculty liaisons or research office personnel, at selected Egyptian universities. We collaborated with them to disseminate the study invitation through departmental email lists or internal communication channels, maximizing reach within academic structures.
- Utilizing Online Institutional Directories: We leveraged university websites that maintain comprehensive researcher profiles, including contact information and research areas. This facilitated the identification of potential participants based on their expertise and institutional affiliation.

### Snowball Sampling

- As we enrolled initial participants, we encouraged them to refer colleagues who might be interested in the study. This snowball sampling technique proved advantageous in expanding our reach and recruiting additional researchers from diverse institutions.

### Selection Criteria

Throughout the recruitment process, we adhered to specific criteria to ensure the participation of qualified individuals:

- Academic Background: Only researchers with demonstrable research experience and affiliation with accredited universities or academic institutions were invited.
- Publication History: While not an exclusionary factor, prioritizing researchers with recent publications helped ensure active engagement within their respective fields.

Having identified the target population, we implemented a multi-pronged recruitment strategy to maximize reach and ensure a representative sample. This strategy encompassed two key avenues:

### Utilizing Social Media Networks

- Targeted Outreach through Facebook Groups: We sought participation by posting study invitations in relevant research-focused Facebook groups boasting high membership across various universities and disciplines. To further refine our reach, we leveraged targeted advertising within Facebook, enabling us to connect with researchers whose specific interests closely aligned with our study topic.
- Leveraging Trusted Networks via WhatsApp Groups: We cultivated fruitful collaborations with faculty members and research coordinators at several universities. Through their established WhatsApp groups for researchers, we disseminated study information, capitalizing on trusted personal networks within academic communities.

### Dissemination through Established Email Channels

- Collaboration with University Mailing Lists: We secured permission from multiple universities to send concise email invitations to their faculty and research staff listservs. This approach broadened our reach, guaranteeing inclusivity of researchers from diverse departments and institutions.

Participant responses were recorded consecutively until the desired sample size was attained.

### Study Tool

We employed a self-administered, pre-designed questionnaire hosted on Google Forms to gather data. The questionnaire investigated participants’ knowledge of ChatGPT and other chatbots, their awareness of current chatbot and AI applications, and their attitudes towards ChatGPT and its potential future uses. The complete questionnaire instrument is available in supplementary file 1.

To ensure a targeted and relevant sample, we adopted a non-probability purposive sampling approach. We began by identifying key academic databases and research communities frequented by our target population (researchers). Subsequently, we implemented a snowball sampling technique, leveraging the recommendations of initial participants to reach additional researchers within these communities.

### Sample Size

Using One-Epi software [14] for sample size calculation, and assuming 50% proportion of having good knowledge about ChatGPT among researchers, the minimum sample size required was 196 subjects at 95% level of confidence and 7% margin of error. A preliminary stage was conducted to assess the validity and reliability of the questionnaire before its wider use. Initially, three Egyptian experts in the field of AI research were asked to evaluate the appropriateness of the questionnaire items and correctly measure the researchers’ knowledge, perception and attitude towards the use of ChatGPT in research. Then, minimal corrections were made. The next step was the pre-testing of the questionnaire. We included 20 participants who were asked to fill out the questionnaire twice, three weeks apart. The collected data were used to assess internal consistency reliability using Cronbach’s alpha as well as test-retest reliability using intra-class correlation coefficient. The results showed adequate internal consistency reliability (With Cronbach’s Alpha = 0.81). Additionally, the intra-class correlation coefficient was 0.96.

### Statistical Analysis

The statistical analysis for this study was conducted using Minitab 17.1.0.0 for Windows (Minitab Inc., 2013, Pennsylvania, USA). Data normality was assessed using the Shapiro-Wilk test. Continuous data were summarized as mean ± standard deviation or median (interquartile range), while categorical data were presented as frequencies and percentages. The correlation between the degrees of disagreement/agreement and the demographic characters was performed using an independent t-test or person correlation coefficient. All tests were two-tailed, with a significance level set at *p* < 0.05.

### IRB Approval

Approval of Institutional Review Board (IRB) at Faculty of Medicine, Alexandria University was obtained before starting the study (IRB No: 00012098).

### Ethical Considerations

Prior to accessing the questionnaire, participants were required to provide online informed consent to participate in the study. All data were collected and presented anonymously, ensuring participant confidentiality throughout the study and beyond.

## Results

A total of 512 researchers were invited to participate in the study through the aforementioned communication tools. Data was collected between June and August 2023. Of those invited, 320 agreed to participate, resulting in a response rate of 64%. However, only 200 (62.5%) individuals ultimately completed the survey. We used the “limit to 1 response” option in Google Forms to ensure participants could only submit the form once. Table 1 presents the demographic characteristics of these final respondents. The majority of participants were female (57.5%), with a median age of 35 years (interquartile range: 30–42 years). Notably, over two-thirds (68%) belonged to the medical sector.

**Table 1:** Demographic and academic data of participants

Factors	Total (*n* = 200)	
	**Median**	**IQR**
Age	35	(30–42)
**Sex**	**N**	**%**
Female Male	115 85	57.5 42.5
**Specialty**	**N**	**%**
Medical	136	68.0
Non-medical	64	32.0
**Affiliation**	**N**	**%**
University or research center	150	75.0
Other	50	25.0
**Academic degree**	**N**	**%**
Master or Doctorate degree	137	68.5
Other	63	31.5
**Publications**	**N**	**%**
No publications	72	36.0
One or more publications	128	64.0
**Number of publications**	**Median**	**IQR**
	3.5	(1–20)

N: number, IQR: inter quartile range, Continuous data presented as median and IQR, and categorical data as number and percentage

Table 2 details our participants’ familiarity with ChatGPT and other chatbots, as well as their engagement with AI tools in their research endeavors. While a substantial majority (67%) had encountered ChatGPT, its uptake within the research setting remained limited, as indicated by the relatively low proportion (11.5%) who reported using it. Similarly, familiarity with other chatbots exceeded usage, with 32% acknowledging awareness and 27.5% reporting prior use. Notably, while nearly half (44%) of the participants identified as familiar with the broad concept of AI, only 20% actively integrated AI applications into their research activities.

**Table 2:** Knowledge and use of ChatGPT and other chatbots by participants

Knowledge items	Total (*n* = 200)	
**Have you heard about Chat-GPT before today?**	N	%
No	66	33.0
Yes	134	67.0
**Have you heard of other chatbots?**	N	%
No	136	68.0
Yes	64	32.0
**Have you ever used chatbots before (e.g.: during customer services)?**	N	%
No	145	72.5
Yes	55	27.5
**Are you familiar with the concept of Artificial Intelligence (AI) in research?**	N	%
No	112	56.0
Yes	88	44.0
**Have you ever used artificial intelligence tools in your research work?**	N	%
No	160	80.0
Yes	40	20.0
**Have you ever used ChatGPT in your research work?**	N	%
No	177	88.5
Yes	23	11.5
What did you use it for? *	N	%
Data analysis	5	2.5
Language proofreading	8	4.0
Looking for references	14	7.0
Rephrasing of paragraphs	21	10.5
Translation of a scientific article or work	4	2.0
Writing part of an article/academic work	9	4.5

Categorical data were presented as numbers and percentage

*Categories are not mutually exclusive, and participants were able to select more than one choice

**Fig. 1:**
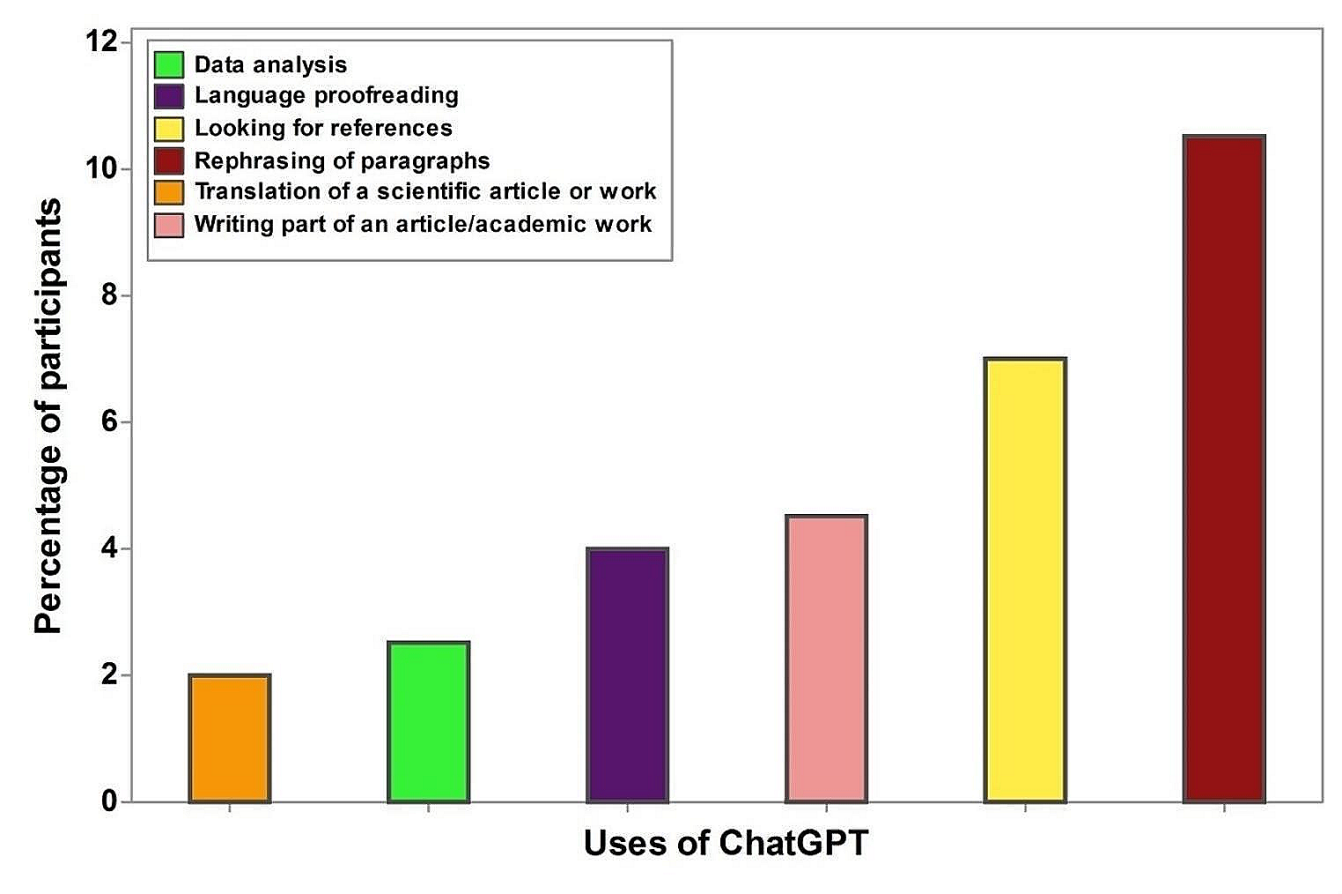
Participant-reported uses of ChatGPT in research activities

Figure 1 illustrates the distribution of ChatGPT usage among participants. Rephrasing paragraphs (10%) and searching for references (7%) emerged as the most prevalent applications, while data analysis and translation assignments constituted the least frequently employed tasks (2.5% and 2%, respectively).

The survey revealed a positive attitude among participants regarding the potential benefits of ChatGPT in research, with a majority agreeing on its usefulness and applicability. Notably, over one-third endorsed the idea of listing ChatGPT as a contributing author in publications. However, concerns surfaced surrounding the potential displacement of researchers’ roles, particularly in tasks like language editing, statistics, and data analysis.

**Fig. 2:**
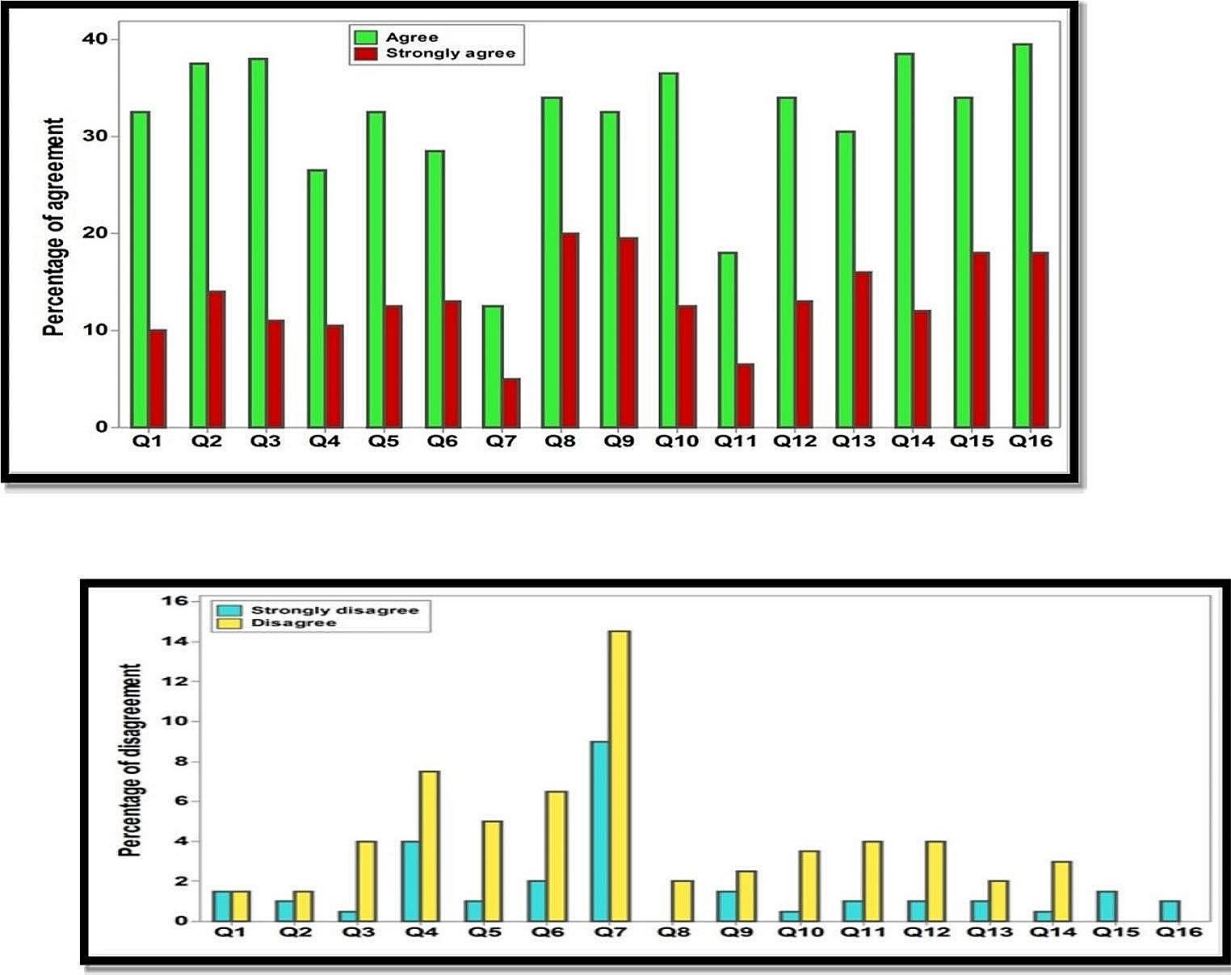
Participants’ agreement and disagreement towards survey statements

Interestingly, approximately half the participants acknowledged the utility of ChatGPT for paraphrasing, resource retrieval, and data analysis, albeit with reservations about the accuracy of its output. They further recognized the potential for enhanced data collection, research efficiency, and productivity if ChatGPT’s capabilities were expanded. Nevertheless, ethical concerns related to AI integration in scientific research resonated with another half of the respondents, highlighting the need for further consideration of these implications (Table 3; Fig. 2 further detail these findings).

**Table 3:** Attitude and views of participants towards uses and future prospect of ChatGPT and other chatbots

Attitude and future prospect	Total (*n* = 200)									
	**Strongly** **disagree**		**Disagree**		**Not sure**		**Agree**		**Strongly** **agree**	
	**N**	**%**	**N**	**%**	**N**	**%**	**N**	**%**	**N**	**%**
1. I am going to use ChatGPT in my future research	3	1.5	3	1.5	109	54.5	65	32.5	20	10.0
2. I think ChatGPT is/ will be useful in academic research.	2	1.0	3	1.5	92	46	75	37.5	28	14.0
3. I think ChatGPT is/will be useful in the peer review of articles.	1	0.5	8	4.0	93	46.5	76	38.0	22	11.0
4. If ChatGPT helps with research, I think it should be listed as an author on scientific publications	8	4.0	15	7.5	103	51.5	53	26.5	21	10.5
5. In the future, I think artificial intelligence will replace the functions of language editors who edit scientific publications	2	1.0	10	5.0	98	49.0	65	32.5	25	12.5
6. In the future, I think artificial intelligence will replace the functions of statisticians and data analyzers	4	2.0	13	6.5	100	50.0	57	28.5	26	13.0
7. In the future, I think artificial intelligence will replace the functions of researchers in general	18	9.0	29	14.5	118	59.0	25	12.5	10	5.0
8. I think ChatGPT is/will be specifically useful for paraphrasing of paragraphs	0	0	4	2.0	88	44.0	68	34	40	20.0
9. I think ChatGPT is/will be specifically useful to search for resources.	3	1.5	5	2.5	88	44.0	65	32.5	39	19.5
10. I think ChatGPT is/will be specifically useful for data analysis	1	0.5	7	3.5	94	47.0	73	36.5	25	12.5
11. I think results generated by ChatGPT are not accurate	2	1.0	8	4.0	141	70.5	36	18.0	13	6.5
12. I think ChatGPT will facilitate medical services in the future (e.g. data collection from patients)	2	1.0	8	4.0	96	48.0	68	34.0	26	13.0
13. I think there are ethical issues associated with the use of ChatGPT in research	2	1.0	4	2.0	101	50.5	61	30.5	32	16.0
14. I think ChatGPT can improve the efficiency and productivity of research	1	0.5	6	3.0	92	46.0	77	38.5	24	12.0
15. I think ChatGPT needs improvement to be more useful in research	3	1.5	0	0.0	93	46.5	68	34.0	36	18.0
16. I think I will learn how to use artificial intelligence in my research work in the future.	2	1.0	0	0.0	83	41.5	79	39.5	36	18.0

Categorical data were presented as numbers and percentages

### Factors Influencing ChatGPT and Chatbot Utilization

Table 4 presents findings from univariate analyses investigating factors related to the use of ChatGPT and other chatbots by the participants. A prominent revelation is the markedly higher inclination towards these tools among younger researchers. Their median age of 34 years (IQR: 28–38) stands in contrast to 37 years (IQR: 30–45) for non-users (*p* = 0.01). Furthermore, awareness of the existence of ChatGPT and other chatbot models significantly influenced their propensity to utilize them (*p* = 0.001). Notably, no statistically significant associations were observed between the use of these technologies and variables like sex, specialty, academic affiliation, degree level, or publication count.

**Table 4:** Factors affecting the use of ChatGPT and other Chatbots

	Use of ChatGPT and other chatbots						
Factors	Yes (*n* = 76)			No (*n* = 124)			
	Median	Q2	Q3	Median	Q2	Q3	p
Age	33.5	28.25	38	36.5	30.25	44.75	**0.01** ^**$**^
Sex	N	%		N	%		
Male	33	43.42		52	41.93		0.46
Female	43	56.57		72	58.06		
Specialty	N	%		N	%		
Medical field	51	67.105		85	68.548		0.95
Non-medical	25	32.895		39	31.452		
Affiliation	N	%		N	%		
University and research center	60	78.947		90	72.581		0.41
Others	16	21.053		34	27.419		
Have you heard about ChatGPT before today?	N	%		N	%		
No	0	0		66	53.226		**0.001** ^*^
Yes	76	100		58	46.774		
Have you heard of other chatbots?	N	%		N	%		
No	25	32.89		111	89.51		**0.001** ^*^
Yes	51	67.11		13	10.48		

N: number, Q2: quartile 2, Q3: quartile 3, the numerical data presented as median and inter quartile range and categorical data as number and percentage, the test of significant was *: Chi square test, and $: Mann Whitney test, *p* < 0.05 considered significant

The multivariate analysis presented in Table 5 identified two key factors influencing the use of ChatGPT and similar models: researcher age and prior familiarity with these models. Younger researchers were found to be slightly more likely to adopt these models for each year younger, while those with prior knowledge were 17 times more likely to do so (odds ratios = 1.1 and 17, p-values = 0.05 and < 0.001, respectively).

**Table 5:** Independent factors that influenced the use of ChatGPT

Factors	OR	95% CI	p
Age	1.1	(0.9362, 1.0030)	**0.05**
Have you heard of other chatbots? (Yes)	17	(7.9644, 36.0965)	**< 0.001**

OR: odd ratio, CI: confidence interval, the test of significant: multiple logistic regression models with stepwise selection models, *p* < 0.05 considered significant

We also correlated the attitude and future uses of ChatGPT with different academic characteristics of participants. The analysis of correlations between participants’ academic background (specialty, affiliation, publications) and their attitudes and future uses of ChatGPT, presented in Supplementary Tables 1–3, yielded no statistically significant relationships.

## Discussion

This study investigated the perceptions of Egyptian researchers regarding the utilization of ChatGPT in academic research. The findings indicate that ChatGPT adoption remains in its nascent stages within this cohort. Despite this, awareness of its potential benefits is burgeoning, with many researchers expressing interest in leveraging it to enhance their work.

Despite the presence of earlier chatbot releases, ChatGPT has sparked a notable surge of interest and engagement within academic circles. This is reflected in the relatively high degree of familiarity with ChatGPT among our study participants compared to other chatbots. Intriguingly, this awareness did not necessarily translate into widespread research utilization. Currently, the primary identified applications of ChatGPT in research involve paragraph rephrasing and reference retrieval. Notably, data analysis emerged as a potential function, although concerns surrounding the accuracy and reliability of outputs were expressed by many participants. Interestingly, our data revealed a positive association between age and chatbot use, with younger researchers exhibiting a higher likelihood of engagement. This association can potentially be attributed to the increased technological fluency and comfort often observed in younger generations, which is further supported by the observed higher usage among participants with prior familiarity with chatbots.

While ChatGPT and other language models present potential benefits for research endeavors, inherent limitations require critical consideration. One primary concern lies in their restricted comprehension of the complex nuances within the published literature. This inadequacy can lead to erroneous analyses and potentially misleading conclusions drawn from the processed information. Furthermore, the absence of robust citation mechanisms within these models creates a significant risk of perpetuating misinformation [15, 16].

This concern is demonstrably illustrated by an anecdotal incident encountered during the present study’s preparation. The first author of this manuscript engaged ChatGPT in a query regarding a specific research topic of interest. While the model provided a seemingly plausible explanation, its attempts at referencing relevant sources proved demonstrably unreliable. The two purported complete references (authors, year, journal, and DOI) offered by ChatGPT were either entirely fabricated, with a DOI linked to an unrelated article, or an existing publication, but its content bore no connection to the initial query, and the accompanying DOI was inaccurate.

While the identified limitations of ChatGPT, particularly its limited understanding of literature and unreliable citation mechanisms, pose significant challenges to its widespread adoption in research, we believe that advancements in AI technology and refined training methodologies hold the potential to mitigate these concerns over time. Nevertheless, until such advancements materialize, researchers and students should exercise caution when utilizing ChatGPT. Rigorous verification of the quality and accuracy of generated outputs is essential, and its application should be restricted to tasks that require minimal literary analysis or citation accuracy. Currently, tasks such as summarizing existing literature, enhancing written content, and conducting basic statistical analyses appear to be more suitable for ChatGPT’s capabilities.

More than one third of participants in our study believed ChatGPT could be designated as an author on scientific publications under the condition of its meaningful contribution to the research work. However, roughly half expressed concerns regarding the ethical implications of integrating AI applications into scientific research. Additionally, concerns pertaining to ethical, legal, and social issues (ELSI) surrounding chatbot implementation in research have been raised, despite existing examples where ChatGPT has been listed as an author in several articles and preprints [10, 17].

Major publishers have adopted a range of responses to the question of ChatGPT’s potential authorship, with some implementing restrictions on listing it as a co-author and others opting for a complete prohibition of its use. Similarly, the use of text generated by ChatGPT within research manuscripts incurs varying degrees of scrutiny, with some publishers imposing outright bans and others permitting its use for stylistic improvements under specific conditions, such as excluding critical tasks like data analysis and interpretation and mandating transparent disclosure of its involvement [18]. A leading plagiarism detection software company has unveiled a novel technology capable of recognizing AI-assisted writing, encompassing texts produced by ChatGPT [19].

The diverse array of responses to ChatGPT’s utilization in research necessitates closer examination. In this context, it is pertinent to consider the International Committee of Medical Journal Editors (ICMJE) guidelines, which stipulate four essential criteria for authorship in scientific publications and academic works: (1) conceptualization and design, (2) data collection, analysis, and interpretation, (3) substantial contribution to writing, drafting, or critical revision of the intellectual content, and (4) final approval of the version intended for publication [20, 21]; Individuals who do not meet the aforementioned criteria should be recognized solely in the acknowledgments section of the publication [21]. Furthermore, all authors bear the collective responsibility to ensure that any concerns regarding the accuracy or integrity of any aspect of the work are appropriately investigated and satisfactorily addressed [22]. Ethical and responsible authorship hinges upon three fundamental pillars: truthfulness, ensuring no falsity or misrepresentation is present; trustworthiness, demanding authors diligently strive to minimize bias; and fairness, upholding objectivity, and impartiality throughout the research process. Accountability, ethical conduct, and independence are further requisites for authors to fulfill their obligations [22, 23].

Based on the established criteria for authorship outlined above, two primary arguments preclude the listing of ChatGPT as an author on scientific publications. Firstly, its capabilities do not align with the aforementioned requirements. Secondly, and more importantly, ChatGPT lacks the capacity to be held accountable for the presented work, a fundamental characteristic for authorship as stipulated by The ICMJE guidelines, which state that “Chatbots (such as ChatGPT) should not be listed as authors because they cannot be responsible for the accuracy, integrity, and originality of the work, and these responsibilities are required for authorship.” [21].

In line with the International ICMJE authorship criteria, the World Association of Medical Editors (WAME)’s recent recommendations on AI-assisted writing explicitly deny Chatbots the status of author. This exclusion stems from their inability to fulfill crucial authorship responsibilities, such as approving the final version before publication, ensuring the work’s integrity and accuracy, comprehending, and legally signing the conflict-of-interest statement. Consequently, WAME emphasizes that authors bear the ultimate responsibility for the accuracy of any material generated by a chatbot and included in their publications [24].

As technological advancements continue, chatbots may gradually acquire the capability to perform more complex research tasks and, consequently, raise the question of their accountability for their actions. This potential scenario necessitates a critical re-examination of authorship guidelines to address the issue and formulate clear recommendations regarding the attribution of authorship in such situations.

The potential for widespread utilization of ChatGPT in research paper drafting also raises significant ethical concerns surrounding the potential for text similarity within papers addressing the same field. This could manifest as high rates of plagiarism flagged by plagiarism detection software. Additionally, the potential designation of ChatGPT as a co-author presents a novel challenge for the research community, sparking debate amongst supporters and opponents [25, 26]. Furthermore, the issue of transparency regarding AI-generated content within research outputs necessitates clear disclosure practices [27].

A systematic review investigating the potential and pitfalls of ChatGPT in healthcare education and research found that concerns surrounding its use were prevalent in over 90% of analyzed publications. These concerns encompassed ethical considerations, copyright and plagiarism issues, lack of originality, inaccurate content, limited knowledge base, incorrect citations, and a propensity for “artificial hallucination” – the generation of misleading or factually incorrect outputs by AI models [28]. This phenomenon of AI-generated “hallucinations,” particularly pronounced in models trained on extensive unsupervised data, underscores the crucial role of human evaluation in ensuring the accuracy and validity of generated content [29].

Artificial intelligence (AI) is increasingly permeating diverse medical fields, exhibiting promising potential in areas such as basic research, disease diagnosis, patient risk identification, drug discovery, and clinical trials [30, 31]. The integration of AI into healthcare raises a multitude of social concerns, with anxieties regarding the potential for AI to replace doctors occupying a prominent space [32]. This concern regarding AI replacing human practitioners is particularly salient for diagnosticians like radiologists and pathologists, whose workflows may be significantly impacted by AI technologies. While complete automation may not be imminent, the rapid advancements in AI necessitate exploration of the timeline for transitioning to semi-autonomous and eventually fully autonomous diagnostic systems [32].

The emergence of advanced language models like ChatGPT raises analogous questions about their potential roles in research. Specifically, their capabilities can blur the lines of traditional researcher functions, leading to concerns about replacing or significantly automating tasks currently performed by human researchers and supporting personnel, such as data analysts and language editors. Interestingly, similar anxieties regarding potential job displacement by AI have surfaced in other fields, notably with programmer concerns surrounding models like Google DeepMind’s AlphaCode. [33, 34] While current evidence suggests that AI is unlikely to entirely replace researchers in the near future, its growing capabilities necessitate a shift in focus from competition to collaboration. Researchers in the coming years should prioritize adapting to and effectively integrating AI into their workflows, rather than viewing it as a threat.

A key limitation of our study lies in the sampling and recruitment methods, as reliance on self-selection introduces the possibility of non-representative data. This potential bias, where only individuals already interested in the topic participated, may limit the generalizability of our findings, and necessitate caution in interpreting them.

## Conclusions and Recommendations

The increasing popularity of chatbots in academic research presents an opportunity to foster responsible and ethical AI integration. Research efforts should prioritize the development of advanced text analysis techniques for identifying fabricated or misleading content, alongside the development of training programs for researchers to effectively utilize chatbots and other AI tools while adhering to ethical principles. Such initiatives can ensure the benefits of AI augmentation without compromising academic integrity.

## Electronic Supplementary Material

Below is the link to the electronic supplementary material.

Supplementary Material 1

Supplementary Material 2

## Data Availability

The datasets used and/or analyzed during the current study are available from the corresponding author on reasonable request.

## Abbreviations

AI: Information Technology
ChatGPT: Chat Generative Pre-Trained Transformer
DOI: Digital Object Identifier
ELSI: Ethical, legal and social issues
ICMJE: International Committee of Medical Journal Editors
