# Supplementary material for: Knowledge, Perceptions and Attitude of Researchers Towards Using ChatGPT in Research
Source: J Med Syst. 2024 Feb 27;48(1):26. doi: 10.1007/s10916-024-02044-4 (PMC10899415; doi:10.1007/s10916-024-02044-4)
Supplement: Supplementary file 1 — Supplementary Material 1 [file 10916_2024_2044_MOESM1_ESM.docx]

**Supplementary files**

**Survey to evaluate the knowledge, perceptions and attitude towards using ChatGPT and other chatbots in academic research**

The data collected from this study will only be used for scientific purposes.
What are chatbots? chatbots are computer program that use artificial intelligence
designed to simulate conversation with human users via text or text-to-speech,
especially over the internet. Chatbots are primarily used in business to offer 24/7
customer support

What is ChatGPT? ChatGPT is an artificial-intelligence (AI) chatbot that has been developed by OpenAI and launched in November 2022. It can have conversations on topics from history to philosophy to scientific topics, and suggest edits to computer programming code. The chatbot has also been used extensively in academic research to assist in writing, to analyze data, and to avoid plagiarism. Some researchers even listed ChatGPT as author on their academic papers.

What is this survey about? This survey is about the evaluation of the knowledge, perception and attitude of researchers towards using ChatGPT and similar chatbots in scientific research.

**Do you agree to participate in this study?**

- Yes
- No

1. **General information about the participant**
2. What is your age in numbers?
3. What is your gender?

- Male
- Female

1. What is your specialty (e.g Medicine, engineering, chemistry..etc)?
2. What is your affiliation?
   - University
   - Research center
   - Governmental organization
   - Non-governmental organization
   - Other
3. What is your highest academic degree?

- Bachelor degree
- Post graduate Diploma
- Master degree
- PhD

1. What is the number of your publications?
2. **Knowledge about ChatGPT and other chatbots**
3. Have you heard of ChatGPT before today?

- Yes
- No

1. Have you heard of other chatbots?

- Yes
- No
- Not sure

1. Have you ever used chatbots before (e.g.: during customer services)?

- Yes
- No

1. Are you familiar with the concept of Artificial Intelligence (AI) in
   research?

- Yes
- No
- Not sure

1. Have you ever used AI-powered tools in your research work?

- Yes
- No
- Not sure

1. Have you ever used ChatGPT in your research work?

- Yes
- No

1. If the answer to the past question is yes, what did you use it for? (Choose all that apply)

- Writing part of an article/academic work
- Rephrasing of paragraphs
- Language proofreading
- Data analysis
- Searching for references
- Translation of a scientific article' or work
- Other (Please specify)
- No applicable

1. **Attitude and future use of ChatGPT and similar chatbots**

|  | Strongly agree | Agree | Not sure | Disagree | Strongly disagree |
| --- | --- | --- | --- | --- | --- |
| 1. I am going to use ChatGPT in my future research |  |  |  |  |  |
| 1. I think ChatGPT is/ will be useful in academic research. |  |  |  |  |  |
| 1. I think ChatGPT is/will be useful in the peer review of articles. |  |  |  |  |  |
| 1. If ChatGPT helps with research, I think it should be listed as an author on scientific publications |  |  |  |  |  |
| 1. In the future, I think artificial intelligence will replace the functions of language editors who edit scientific publications |  |  |  |  |  |
| 1. In the future, I think artificial intelligence will replace the functions of statisticians and data analyzers |  |  |  |  |  |
| 1. In the future, I think artificial intelligence will replace the functions of researchers in general |  |  |  |  |  |
| 1. I think ChatGPT is/will be specifically useful for paraphrasing of paragraphs |  |  |  |  |  |
| 1. I think ChatGPT is/will be specifically useful to search for resources. |  |  |  |  |  |
| 1. I think ChatGPT is/will be specifically useful for data analysis |  |  |  |  |  |
| 1. I think results generated by ChatGPT are not accurate |  |  |  |  |  |
| 1. I think ChatGPT will facilitate medical services in the future (e.g data collection from patients) |  |  |  |  |  |
| 1. I think there are ethical issues associated with the use of ChatGPT in research |  |  |  |  |  |
| 1. I think ChatGPT can improve the efficiency and productivity of research |  |  |  |  |  |
| 1. I think ChatGPT needs improvement to be more useful in research |  |  |  |  |  |
| 1. I think I will learn how to use artificial intelligence in my research work in the future. |  |  |  |  |  |

Thank you for sharing your opinions. We appreciate your participation!

**Supplementary table 1: Correlation between the attitude and future uses of ChatGPT and participants' specialty**

|  | **Specialty** | **Mean** | **Std. Deviation** | **Significance** |
| --- | --- | --- | --- | --- |
| Q1 | Medical | 0.5000 | 0.74037 |  |
|  | Non-medical | 0.4375 | 0.79433 | 0.936 |
| Q2 | Medical | 0.6471 | 0.75552 |  |
|  | Non-medical | 0.5625 | 0.83333 | 0.678 |
| Q3 | Medical | 0.5515 | 0.73857 |  |
|  | Non-medical | 0.5469 | 0.81513 | 0.425 |
| Q4 | Medical | 0.3456 | 0.92998 |  |
|  | Non-medical | 0.2656 | 0.85898 | 0.270 |
| Q5 | Medical | 0.4926 | 0.81646 |  |
|  | Non-medical | 0.5313 | 0.81589 | 0.919 |
| Q6 | Medical | 0.3897 | 0.86215 |  |
|  | Non-medical | 0.5469 | 0.88962 | 0.502 |
| Q7 | Medical | -0.1471 | 0.93118 |  |
|  | Non-medical | 0.0000 | 0.85449 | 0.159 |
| Q8 | Medical | 0.7059 | 0.78048 |  |
|  | Non-medical | 0.7500 | 0.85449 | 0.168 |
| Q9 | Medical | 0.6471 | 0.83026 |  |
|  | Non-medical | 0.6875 | 0.95743 | 0.216 |
| Q10 | Medical | 0.5809 | 0.75577 |  |
|  | Non-medical | 0.5469 | 0.81513 | 0.653 |
| Q11 | Medical | 0.1985 | 0.61848 |  |
|  | Non-medical | 0.3594 | 0.78411 | 0.07 |
| Q12 | Medical | 0.5588 | 0.79596 |  |
|  | Non-medical | 0.5000 | 0.83571 | 0.750 |
| Q13 | Medical | 0.5735 | 0.83117 |  |
|  | Non-medical | 0.6094 | 0.78916 | 0.840 |
| Q14 | Medical | 0.5956 | 0.72397 |  |
|  | Non-medical | 0.5625 | 0.83333 | 0.229 |
| Q15 | Medical | 0.6691 | 0.77989 |  |
|  | Non-medical | 0.6719 | 0.90947 | 0.235 |
| Q16 | Medical | 0.7574 | 0.75519 |  |
|  | Non-medical | 0.6875 | 0.85217 | 0.125 |

**Supplementary table 2: Correlation between the attitude and future uses of ChatGPT and participants' affiliation**

|  | Research center and university | Mean | Std. Deviation | Significance |
| --- | --- | --- | --- | --- |
| Q1 | University or research center | 0.5067 | 0.76629 |  |
|  | Others | 0.4000 | 0.72843 | 0.344 |
| Q2 | University and research center | 0.6333 | 0.81444 |  |
|  | Others | 0.5800 | 0.67279 | 0.165 |
| Q3 | University and research center | 0.5800 | 0.77086 |  |
|  | Others | 0.4600 | 0.73429 | 0.615 |
| Q4 | University and research center | 0.3600 | 0.93608 |  |
|  | Others | 0.2000 | 0.80812 | 0.065 |
| Q5 | University and research center | 0.5133 | 0.84135 |  |
|  | Others | 0.4800 | 0.73512 | 0.254 |
| Q6 | University and research center | 0.4267 | 0.91480 |  |
|  | Others | .4800 | 0.73512 | 0.137 |
| Q7 | University and research center | -0.0867 | 0.91179 |  |
|  | Others | -0.1400 | 0.90373 | 0.905 |
| Q8 | University and research center | 0.7400 | 0.80627 |  |
|  | Others | 0.6600 | 0.79821 | 0.987 |
| Q9 | University and research center | 0.7000 | 0.84940 |  |
|  | Others | 0.5400 | 0.93044 | 0.701 |
| Q10 | University and research center | 0.5800 | 0.79655 |  |
|  | Others | 0.5400 | 0.70595 | 0.310 |
| Q11 | University and research center | 0.2133 | 0.66137 |  |
|  | Others | 0.3600 | 0.72168 | 0.108 |
| Q12 | University and research center | 0.5333 | 0.84874 |  |
|  | Others | 0.5600 | 0.67491 | 0.082 |
| Q13 | University and research center | 0.6067 | 0.85056 |  |
|  | Others | 0.5200 | 0.70682 | 0.115 |
| Q14 | University and research center | 0.6000 | 0.79427 |  |
|  | Others | 0.5400 | 0.64555 | 0.104 |
| Q15 | University and research center | 0.7067 | 0.79888 |  |
|  | Others | 0.5600 | 0.88433 | 0.723 |
| Q16 | University and research center | 0.7667 | 0.79779 |  |
|  | Others | 0.6400 | 0.74942 | 0.895 |

**Supplementary table 3: Correlation between the attitude and future uses of ChatGPT and academic publications of participants**

|  | Publications | Mean | Std. Deviation | Significance |
| --- | --- | --- | --- | --- |
| Q1 | Publications | 0.5000 | 0.72089 |  |
|  | No publication | 0.4444 | 0.82032 | 0.447 |
| Q2 | Publications | 0.5703 | 0.80043 |  |
|  | No publication | 0.7083 | 0.73996 | 0.601 |
| Q3 | Publications | 0.5313 | 0.75229 |  |
|  | No publication | 0.5833 | 0.78274 | 0.540 |
| Q4 | Publications | 0.3125 | 0.84881 |  |
|  | No publication | 0.3333 | 1.00702 | 0.291 |
| Q5 | Publications | 0.4453 | 0.81142 |  |
|  | No publication | 0.6111 | 0.81458 | 0.902 |
| Q6 | Publications | 0.4375 | 0.87619 |  |
|  | No publication | 0.4444 | 0.87031 | 0.741 |
| Q7 | Publications | -0.1094 | 0.92417 |  |
|  | No publication | -0.0833 | 0.88413 | 0.654 |
| Q8 | Publications | 0.6797 | 0.80288 |  |
|  | No publication | 0.7917 | 0.80382 | 0.944 |
| Q9 | Publications | 0.6250 | 0.87844 |  |
|  | No publication | 0.7222 | 0.85945 | 0.712 |
| Q10 | Publications | 0.5391 | 0.80258 |  |
|  | No publication | 0.6250 | 0.72067 | 0.338 |
| Q11 | Publications | 0.2578 | 0.65504 |  |
|  | No publication | 0.2361 | 0.72176 | 0.387 |
| Q12 | Publications | 0.5547 | 0.83060 |  |
|  | No publication | 0.5139 | 0.76900 | 0.317 |
| Q13 | Publications | 0.6172 | 0.80472 |  |
|  | No publication | 0.5278 | 0.83872 | 0.785 |
| Q14 | Publications | 0.5625 | 0.78118 |  |
|  | No publication | 0.6250 | 0.72067 | 0.508 |
| Q15 | Publications | 0.7031 | 0.83581 |  |
|  | No publication | 0.6111 | 0.79710 | 0.665 |
| Q16 | Publications | 0.7969 | 0.79723 |  |
|  | No publication | 0.6250 | 0.75875 | 0.741 |
